# Parents' use of harsh punishment and young children's behaviour and achievement: a longitudinal study of Jamaican children with conduct problems

**DOI:** 10.1017/gmh.2018.21

**Published:** 2018-10-12

**Authors:** H. Baker-Henningham, T. Francis

**Affiliations:** 1 School of Psychology, Bangor University, Bangor, LL57 2AS, UK; 2 Caribbean Institute for Health Research, University of the West Indies, Kingston 7, Jamaica

**Keywords:** Child behaviour, aetiology, harsh punishment, low- and middle-income countries, school achievement, violence

## Abstract

**Introduction.:**

Harsh punishment by parents is common in low- and middle-income countries (LMIC), yet there is limited evidence from LMIC of the effects of harsh punishment on child outcomes.

**Methods.:**

A longitudinal, prospective study was conducted with children with conduct problems to examine the associations between parents’ use of harsh punishment during the preschool years on child behaviour and school achievement in grade one of primary school. As part of an efficacy trial in 24 preschools, 225 children with the highest level of teacher-reported conduct problems were evaluated and their parents reported on how often they used harsh punishment. Outcome measures in grade one included child conduct problems by independent observation, teacher and parent report, child social skills by teacher and parent report, direct tests of children's academic achievement and language skills, and tester ratings of child attention and impulse control.

**Results.:**

Children had a mean age of 6.92 years and 61% were boys. All parents reported using harsh punishment. After controlling for child age and sex, socio-economic status, parents’ involvement with child and maternal education, frequency of harsh punishment was associated with growth in child conduct problems by independent classroom observations (*p*  =  0.037), parent (*p*  =  0.018) and teacher (*p*  =  0.044) report, a reduction in child social skills by teacher (*p*  =  0.024) and parent (*p*  =  0.014) report and poorer attention during the test session (*p*  =  0.049).

**Conclusion.:**

The associations between frequency of parents’ use of harsh punishment with their preschoolers with conduct problems and later child behaviour indicate a need to train parents in non-violent behaviour management.

## Background

Violence against children is a global public health problem and is highly prevalent in low- and middle-income countries (LMIC). Violence against children within the family is one of the most common forms of child maltreatment and often occurs due to harsh punishment methods being used to discipline children (UNICEF, 2010). Parent discipline practices can be classified into three main categories: (1) non-violent (e.g. removing privileges, explaining why something is wrong), (2) psychologically aggressive (e.g. name-calling, yelling) and (3) physically violent (e.g. slapping, beating with an object). Harsh punishment includes the use of psychologically aggressive and physically violent practices and these are considered violence against children or child maltreatment (Straus *et al*. 1998).

Harsh punishment in childhood is associated with multiple negative outcomes, which persist into adulthood. In a meta-analytical review of 88 studies, Gershoff (2002) found that corporal punishment was associated with increased aggression [effect size (ES): 0.36], poor mental health (ES = −0.49), negative parent–child relationships (ES = −0.58) and delinquent and antisocial behaviour (ES  =  0.42) in childhood. Effects of corporal punishment persisted into adulthood leading to increased aggression (ES  =  0.57), poor mental health (ES = −0.09), criminality and antisocial behaviour (ES  =  0.42) and an increased risk for perpetrating child or spouse abuse (ES  =  0.13) (Gershoff, 2002). A more recent meta-analytical review of 124 studies found significant associations between child physical and emotional abuse and depression [physical abuse: odds ratio (OR)  =  1.54, emotional abuse: OR = 3.06), drug use (physical abuse: OR = 1.92, emotional abuse: OR = 1.41), suicide attempts (physical abuse: OR = 3.40, emotional abuse: OR = 3.37) and sexually transmitted infections and risky sexual behaviour (physical abuse: OR = 1.78, emotional abuse: OR = 1.75) (Norman *et al*. 2012). Studies examining associations between harsh punishment and academic achievement show mixed results (Romano *et al*. 2015). There is some evidence that the effect of harsh punishment on achievement is moderated by risk status with more vulnerable children at particular risk, for example, children in out-of home care (Romano *et al*. 2015) and children with HIV (Sherr *et al*. 2016).

Theoretical and empirical models of the mechanisms through which harsh punishment affects child outcomes are numerous and include observational learning, social control theory, social informational processing theory and emotional and sensory arousal (Gershoff, 2002). The models share a common theme, which is that the effect of harsh punishment on child outcomes is mediated through its effects on children's internal cognitive and affective processes (Gershoff, 2002; Straus & Paschall, 2009; Maguire-Jack *et al*. 2012). That is, harsh punishment is hypothesised to affect children's thoughts and emotions which in turn influences their behaviour and makes them more susceptible to further negative experiences and/or interactions from others, including parents, teachers and peers.

In the Jamaican context, the use of harsh punishment as a disciplinary strategy is pervasive at home and at school. In the UNICEF Multiple Indicator Cluster Study, 84% of caregivers of 2–4 years old children reported that they or someone in their household had used physical violence with their child in the past month while 71% had used psychological aggression (Lansford & Deater-Deckard, 2012). In a study of 1300 children in grade five of primary school, 92.5% of children had been reported being physically punished at school during the school year and exposure to physical punishment was associated with children's academic achievement in a dose–response manner: maths (high exposure: ES = −0.23; moderate exposure: ES = −0.09), reading (high exposure: ES = −0.44; moderate exposure: ES = −0.17) and spelling (high exposure: ES = −0.24; moderate exposure: ES = −0.14) (Baker-Henningham *et al*. 2009). A further study of 1171 children aged 11–12 years reported a lifetime experience of verbal aggression and physical violence of 97.2% at home and 86.2% at school and poly-victimisation (including harsh punishment, exposure to community violence and witnessing domestic violence) was associated with poor intellectual functioning and for boys, increased behavioural risk (Samms-Vaughan & Lambert, 2017).

In this study, we investigate the associations between harsh punishment and later child outcomes for young children with high levels of conduct problems. Harsh punishment is considered a causal risk factor not only for the development, but also for the persistence of child behaviour problems (Jaffee *et al*. 2012). The prevalence of conduct problems in children ranges from 7% to 25% and children with conduct problems are at heightened risk for developing disruptive behaviour disorders in later childhood (Webster-Stratton & Hammond, 1998). Disruptive behaviour disorders (encompassing conduct disorder and oppositional deviant disorder), are one of the most common forms of child psychopathology with a worldwide prevalence estimate of 5.7% (Polanczyk *et al*. 2015). They predict negative outcomes across the lifespan including continued antisocial behaviour, school failure and involvement in crime and violence (Moffitt & Scott, 2008). In Jamaica, 12% of 5–6 years old children were identified as having externalizing disorders and none of these children had accessed mental health services (Samms-Vaughan, 2005). Furthermore, in a study in 24 inner city Jamaican preschools, 21% (364/1733) of 3–6 years old children had four or more symptoms of conduct problems as reported by the teacher (Baker-Henningham *et al*. 2012).

We are aware of no longitudinal studies with prospective measures of parent discipline and child behaviour from Jamaica or the wider Caribbean and the majority of studies in LMIC involve older children with limited evidence of the effect of harsh punishment in the early childhood years. Early childhood is a particularly sensitive period, and experiences in early childhood have long-term effects on brain function, cognition and psychosocial functioning thus providing the foundation for future physical and mental health (Walker *et al*. 2011; Black *et al*. 2016). We hypothesised that the frequency of parents’ use of harsh punishment, defined as psychologically and physically violent discipline practices, during the preschool years with disadvantaged children with high levels of conduct problems would be associated with: (1) worsening behaviour over time including increased conduct problems and decreased social skills at school and at home between preschool and grade one of primary school and (2) poorer academic achievement, oral language and self-regulation skills in grade one of primary school.

## Methods

### Sampling

Data for this paper were collected as part of a follow-up study of a previously conducted efficacy trial in 24 community preschools situated in disadvantaged areas of Kingston, Jamaica (Baker-Henningham *et al*. 2012). The sampling procedure for the preschools involved surveying all preschools in a specified geographical location to determine the number of classes and number of children per class and selecting preschools that fit the following inclusion criteria: (1) consisted of 3–4 classes of children, (2) had a minimum of 20 children per class and (3) all teachers consented to participate in the trial. Fifty preschools were assessed for eligibility: 25 preschools had too few children per class and/or did not have 3–4 classes and one preschool refused to participate. All classrooms in each preschool were included in the study giving a total of 73 classrooms. Children at high risk of developing conduct problems were selected in preschool and were evaluated as part of the efficacy trial. In the summer term, teachers in the 24 preschools rated all children in their class on a 10-question screen for conduct problems using a four-point scale. Questions were based on age-appropriate items for a diagnosis of conduct disorder from the ICD-10 Classification of Mental and Behavioural Disorders: Diagnostic Criteria for Research (WHO, 1993) and included: loses temper, back chats, disobedient/breaks rules, annoys others, blames others, easily annoyed, often angry, spiteful to others, fights or bullies and destroys property. The responses were summed and the three children scoring the highest on the screen from each class were selected. Six children were kept back in their previous class after the summer holiday and these children were retained in the study and additional children enrolled (Baker-Henningham *et al*. 2012). A total of 225 children were recruited and 214 of these children were tracked into primary school. Three parents were unable to be contacted for interview; the final sample included 211 children in primary school.

**Fig. 1.**
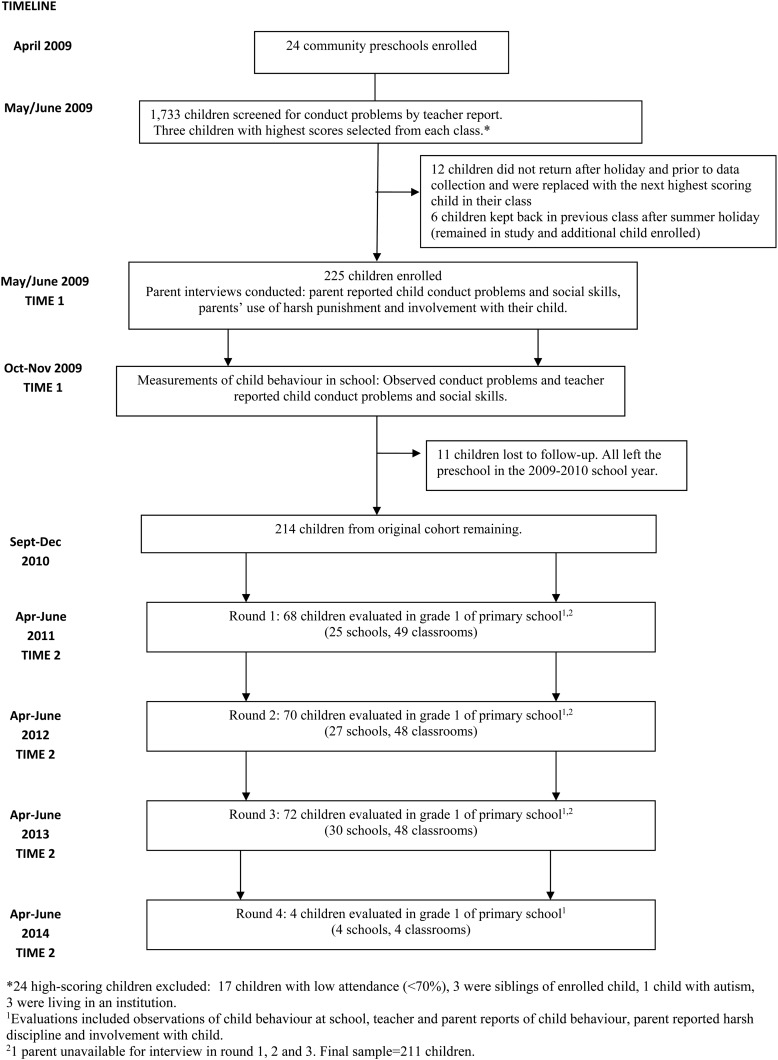
Study profile.

This paper includes data collected at two time points: (i) on enrolment, when the child was in preschool (time 1) and (ii) in the final term of grade one in primary school (time 2). Jamaican preschools cater to children aged 3–6 years and children transition to primary school at age 6. We enrolled children from all classes in preschool, and hence the outcome data in grade one were collected over 4 separate years until all of the children had transitioned to primary school. Data were collected in a total of 50 primary schools and 149 classrooms. See Fig. 1 for the study profile.

The study was approved by the University of the West Indies ethics committee, and written informed consent was obtained from all school principals, teachers and parents in the study. Child assent was not obtained due to the young age of the children in the study (Wendler, 2006).

### Measures

All data were collected as part of an intervention trial, and outcomes across multiple informants in school and home environments were used to evaluate intervention effectiveness (Scott, 2001). Baseline measures of child behaviour were conducted when the children were in preschool and included conduct problems by independent observation, teacher and parent report and social skills by teacher and parent report. Parents also reported on their use of harsh punishment and their involvement with their child at time 1. Baseline measures of child behaviour at home and parents’ use of harsh punishment were conducted in the summer term (May/June) and the measures of child behaviour at school were conducted in the autumn term (October/November). Outcome measures at time 2 included conduct problems by independent observation, teacher and parent report, social skills by teacher and parent report, academic achievement and language skills by direct testing and self-regulation by tester ratings of children's behaviour during the test session (Table 1). All outcome measures were conducted in the summer term of grade one of primary school.

**Table 1.** Child assessments conducted in preschool and/or primary school.

Child development	Measures used
***Measures conducted at time 1 (preschool) and time 2 (primary school)***	
**Conduct problems**	**Factor score of 6 variables used in analysis**
Observed conduct problems^a^	Observations over a total of 12 5-min observation periods (totalling 1 h of observation), including event sampling of aggressive/destructive behaviour, scan sampling of disruptive behaviour and four seven-point rating scales: antisocial behaviour, activity level, on-task behaviour and follows rules and expectations
Teacher-reported conduct problems	Sutter–Eyberg Child Behaviour Inventory (SESBI) frequency scale (Rayfield *et al*. 1998)
Parent-reported conduct problems	Eyberg Child Behaviour Inventory (ECBI) frequency scale (Eyberg & Ross, 1978)
**Social skills**	
Teacher-reported social skills	Prechool and Kindergarten Behaviour Scales (PKBS): Social Skills Scale (Merrell, 1996) at time 1
	School Social Behaviour Scales 2 (SBSS-2): Social Competence Scale (Merrell, 2002) at time 2
Parent-reported social skills	Strengths and Difficulties Questionnaire: Prosocial Scale (Goodman, 1999)
***Measures conducted at time 2 only***	
**Academic Achievement**^b^	**Factor score of reading, maths and spelling tests used in analysis**
Reading	Letter word identification and Passage Comprehension subscales from the Woodcock–Johnson III Tests of Achievement (Woodcock *et al*. 2001)
Maths	Calculation and Reasoning and Concepts subscales of the Woodcock–McGrew–Werder Mini-Battery of Achievement (Woodcock *et al*. 1994)
Spelling	Spelling subscale from the Woodcock–Johnson III Tests of Achievement (Woodcock *et al*. 2001)
**Oral language skills^b^**	**Composite of sum of standardised raw scores of both scales used in analysis**
Receptive and expressive language	Understanding Directions and Story Recall from the Woodcock–Johnson III Tests of Achievement (Woodcock *et al*. 2001)
**Self-regulation**	Rated during the test session using 10 4-point scales from the Preschool Self Regulation Assessment: Assessor Report (Smith-Donald *et al*. 2007)
Attention	Five items: pays attention, careful, concentrates, daydreams, distracted
Impulse control	Five items: thinks and plans, refrains from touching test materials, does not interrupt tester, difficulty waiting, remains in seat
**Parenting**	**Measures used**
***Measures conducted at time 1***	
Harsh punishment^a^	The scale included seven questions, answered on a five-point frequency scale: four questions on physical violence and three questions on psychological aggression: (1) slap on bottom, hand or leg, (2) hit with a hard object, e.g. brush, belt, stick, (3) pinch, (4) slap child on the head, face or ears, (5) cuss bad words at him/her, (6) call child names, e.g. idiot, dummy, stupid and (7) say you will send him/her away. The scores were summed to give an overall score ranging from 0 to 28
Involvement with child	The scale included eight questions, answered on a five-point frequency scale: (1) reading storybooks, (2) playing outside with child, (3) playing inside with child, (4) talking with child about school or friends, (5) sit with child as they write, colour or draw, (6) take the child out (e.g. to market), (7) involve child while doing chores and (8) spend 30 min or more doing something fun with the child. Responses were summed to give an overall score ranging from 0 to 32

a Full details of assessment is given in text.

b For the child achievement and language tests, the raw scores were used, rather than the grade based norms, as the tests have not been normed in Jamaica.

All questionnaires were interviewer administered. Eleven research assistants collected the outcome data for this study. Two research assistants administered the parent interviews, three conducted teacher interviews, three conducted child tests and three conducted child observations. Inter-rater reliabilities were calculated between the trainer and data collectors for all measures during training and for a minimum of 10% of all measures during the study. For the questionnaires and child tests, inter-interviewer/tester intraclass correlation coefficients (ICCs) were >0.95. Details of inter-observer reliabilities for observations are provided below. The parent interview was conducted with the primary caregiver, defined as the mother if she spent a minimum of four nights a week with the child. Parent interviews were conducted at the parents’ home; teacher interviews and child tests were conducted at school. All measures were conducted by research staff blind to the study hypothesis and design.

Table 1 provides information of the measures used at each time point. All outcome measures had good internal consistency (Cronbach's *α*: mean  =  0.86, range 0.71–0.97) and test–retest reliability over 2 weeks (ICC: mean  =  0.90, range 0.75–0.99). Full details of the psychometric properties of the measures are given in web tables 1 and 2. Further details of the measures of observed conduct problems and parents’ use of harsh punishment are provided below.

### Observed conduct problems

#### Preschool

Within each class, three children were observed for 5 min each on a rotational basis for a total of 15 min per day per child over 4 days to give a total of 1 hr of observation. Event sampling was used to record aggressive/disruptive behaviours (e.g. hitting, throwing objects) and expressed as frequency per hour. Instantaneous sampling (i.e. recording whether or not the behaviour occurred at each sample point) was used to code disruptive behaviours (e.g. out of seat, shouting) at 15-s intervals, with a maximum possible score of 240. The aggressive/destructive and disruptive behaviours chosen were based on the Dyadic Parent–Child Interaction Coding System (DPICS) (Eyberg & Robinson, 1981) and Multi-Option Observation System for Experimental Studies (MOOSES) (Tapp *et al*. 1995) behaviour categories, operationalised for the Jamaican context. All behaviours were defined in a manual. In addition to the measures of aggressive and disruptive behaviours described above, at the end of each 5-min period, observers rated child behaviour on four seven-point rating scales (see Table 1). Higher scores indicate higher levels of the behaviours. The mean score over 12 5-min intervals for each rating scale was used in the analyses. Observations were conducted by four research assistants and inter-observer reliabilities (ICCs) were: median 0.83 (0.67–0.91) during training and 0.83 (0.67–0.91) during the study.

#### Primary school

The observation schedule in primary school used the same measures as those described above except that observations were conducted over 2 school days for 30 min each day (rather than over 4 school days for 15 min each day). When there was more than one target child in the class, children were observed in 5-min intervals on a rotational basis with either two or three children being observed during each observation period until each child had been observed for a total of 30 min. This was then repeated on day 2. When there was only one target child per class, children were observed for 5 min out of every 10–15 min. Observations were conducted by three research assistants with only one observer present in a classroom at a time. Inter-observer reliabilities were calculated for 5-min observation intervals and the ICCs were median 0.93 (range 0.90–0.95) during training and [0.93 (0.84–0.97)] during the study.

### Parents’ use of harsh punishment

Harsh punishment was defined as the use of psychologically and physically violent discipline practices and was measured using items adapted from the Parent–Child Conflict Tactics Scale (CTSPC) (Straus *et al*. 1998). The psychological aggression, corporal punishment and physical abuse subscales of the CTSPC were piloted with mothers of children who were not in the study to ensure they were appropriate and understandable. After piloting, several questions were adapted and two items were removed as they were considered too offensive to parents (and could lead to reduced cooperation in the intervention trial). The final scale included four physically violent behaviours and three psychologically aggressive responses. Parents were asked if there was anyone else in the home who disciplines their child regularly, and if so, their name was included in the question – e.g. ‘Do you (or grandma) ever pinch (child's name)?’ Parents were then asked whether they used each form of punishment and if so, how often they had used it in the past month. Scores ranged from 0  =  no, 1  =  not in the past month, 2  =  a few times in the past month, 3  =  once or twice a week and 4  =  almost every day. Internal reliability of the scale was adequate (*α* = 0.64).

### Analysis

Multilevel multiple regression analyses were used to determine the association between frequency of parents’ use of harsh punishment on child outcomes in grade one of primary school, controlling for child age and sex, socio-economic status (SES), mothers’ involvement with child, maternal education and baseline score where available. Multilevel multiple regression models are the appropriate form of analysis for clustered data where outcomes are observed at level 1 (children), who are taught in classrooms (level 2), nested in schools (level 3). Random intercept models were used. The dependent variables were child conduct problems by observation, teacher and parent report, child social skills by teacher and parent report, child academic achievement and oral language skills by direct testing and tester ratings of child attention and child impulse control. In all analyses, child age and sex, SES and mothers’ involvement with child at time 1 (preschool), maternal education, dummy variables to control for data collector, intervention group, baseline score (for measures of child conduct problems and social skills at home and at school) and parents’ use of harsh punishment at time 1 were entered as fixed effects, and school and classroom were entered as random effects. All multilevel analyses were conducted in MLWin (version 2.10) (Rasbash *et al*. 2009).

Exploratory factor analysis with varimax rotation was used to reduce the number of outcome variables with separate factor analyses conducted for child observed behaviour, academic achievement and self-regulation during the test. One factor was produced from the observed child behaviour variables (web Table 3) and the academic achievement tests (web Table 4). Factor analysis of the rating scales from the Preschool Self-Regulation Assessment (PSRA), using varimax rotation, produced two factors: a factor reflecting child attention and a factor reflecting child impulse control (web Table 5). The factor scores for these four outcome variables [(1) observed conduct problems, (2) academic achievement, (3) attention and (4) impulse control) were saved as regression scores in SPSS (v. 23) and used in the analyses (DiStefano *et al*. 2009). Teacher and parent-reported child conduct problems and social skills were each measured using a single scale and the raw score of each scale was used in the analyses. The scores of the oral language scales (following direction and story recall) were standardised and summed to give a measure of child oral language skills (Woodcock *et al*. 2001).

Covariates were computed as follows. A factor score of three variables measured in preschool, number of possessions, sanitation and crowding, was used as a measure of SES. Mothers were asked if they had completed primary school, middle school or high school (Table 1), and maternal education was coded as a binary variable representing completed high school or not (0  =  no, 1  =  yes). Dummy variables were created to represent tester, observer, parent interviewer and teacher interviewer at time 2. These variables were entered as control variables in all regressions.

## Results

### Sample characteristics

Children had a mean age of 6.9 years and over 60% were boys (Table 2). The mother was the primary caregiver for 79% of children. A relatively high proportion of these children were currently in the clinical range for conduct problems by teacher and parent report (approximately one-third of the sample). These children were selected because they had the highest levels of behaviour problems during the preschool years (nearly 55% were in the clinical range for conduct problems by teacher report in preschool). Children had a mean age of 4.24 years when baseline assessments were conducted. Raw scores on all outcome measures are given in Table 3.

**Table 2.** Child and caregiver characteristics at time 1 (preschool) and time 2 (grade 1 of primary school).

	Preschool: all classes	Primary school: grade 1
*Child characteristics*		
Child age, years [mean (s.d.)]	4.24 (0.87)	6.92 (0.36)
Number (%) boys	138 (61.3)	–
Clinical range for conduct problems by teacher report^a^, *n* (%)	123 (54.7)	72 (33.8)
Clinical range for conduct problems by parent report^b^, *n* (%)	77 (34.2)	69 (32.7)
Clinical range for conduct problems at home and at school^c^, *n* (%)	45 (20.0)	27 (12.9)
Caregiver characteristics		
Caregiver: mother, *n* (%)	172 (81.5)	166 (78.7)
Father, *n* (%)	16 (7.1)	18 (8.5)
Grandmother, *n* (%)	10 (4.7)	8 (3.8)
Other relative, *n* (%)	13 (5.8)	19 (9.0)
Age of caregiver, years [mean (s.d.)]	30.74 (9.74)	33.39 (9.61)
Number of possessions [mean (s.d.)]^d^	8.89 (2.52)	8.55 (2.45)
Crowding [mean (s.d.)]^e^	2.08 (1.16)	1.86 (1.07)
Sanitation [mean (s.d.)]^f^	9.47 (2.73)	9.82 (2.48)
Caregiver employed, *n* (%)	86 (40.8)	125 (59.2)
Caregiver education: primary school only, *n* (%)	1 (0.4)	–
Completed middle school, *n* (%)	131 (58.2)	
Completed high school, *n* (%)	93 (41.3)	
Caregiver practices		
Harsh punishment [mean (s.d.)]^g^	8.32 (4.84)	–
Involvement with child [mean (s.d.)]^h^	20.20 (5.28)	–

a Above cut-off (>150) on Sutter–Eyberg Student Behaviour Inventory (SESBI) intensity scale.

b Above cut-off (>130) on Eyberg Child Behaviour Inventory (ECBI) intensity scale.

c Above cut-off on SESBI and ECBI intensity scales.

d Number of possessions from a list of 15 items: stove, fridge, washing machine, sofa or soft chair, mobile telephone, landline, radio, CD player, TV, Cable TV, DVD player, computer, bicycle, motorbike, motor car.

e Number of people per room.

f Type of toilet and water supply.

g Min score  =  0, max score  =  28.

h Min score  =  0, max score  =  32.

**Table 3.** Raw scores of child outcomes measured in grade 1 of primary school.

	Subsample (*n* = 211)
Structured observations of child behaviour	Median (range)
Aggressive/destructive behaviour^a^	7 (0–40)
Disruptive behaviour^b^	26 (0–99)
Observer rating scales of child behaviour^c^	Mean (s.d.)
Conduct problems	2.12 (0.71)
Activity level	2.88 (0.34)
Follows rules and expectations	5.27 (0.70)
On-task behaviour	4.86 (0.95)
Teacher reports of child behaviour	Mean (s.d.)
Conduct problems (SESBI)	133.85 (45.72)
Social skills (SSSBS-2)	101.40 (22.28)
Parent-reported child behaviour	Mean (s.d.)
Conduct problems (ECBI)	119.59 (25.29)
Social skills (SDQ)	8.00 (1.93)
Child academic tests	Mean (s.d.)
Letter word identification^d^	23.05 (8.92)
Reading comprehension^d^	9.92 (4.75)
Spelling^d^	18.16 (4.65)
Math calculation, median (range)^d^	5.00 (0–12)
Math reasoning, median (range)^d^	25.00 (0–31)
Following directions^d^	19.02 (9.01)
Story recall^d^	22.15 (14.60)
Tester ratings of child behaviour during academic test session	Median (range)
Child attention^e^	13.00 (2–15)
Child impulse control^f^	13.00 (2–15)

SESBI, Sutter–Eyberg School Behavior Inventory; SSBS, Student School Behaviour Scales; SDQ, Strengths and Difficulties Questionnaire; ECBI, Eyberg Child Behavior Inventory.

a Event sampling: number of events in 1 h.

b Instantaneous sampling at 15 s intervals over a total of 1 h (max possible score  =  240).

c Mean of 12 ratings conducted every 5 min on a seven-point scale, where 0 is low and 7 is high.

d Raw scores are presented and were used in the analyses.

e Sum of five ratings on a four-point scale (0–3): not distracted, pays attention, sustains concentration, does not daydream and careful.

f Sum of five ratings on a four-point scale (0–3): does not interrupt, refrains from touching, thinks and plans, no difficulty waiting, remains in seat.

### Parents’ use of harsh punishment

All parents reported using harsh punishment with their child when they were in preschool (time 1) and scores ranged from 1 to 23. The most commonly used punishment was slapping on the bottom, hand, arm or leg with approximately 50% of parents reporting slapping their child at least once a week in the past month (Table 4).

**Table 4.** Parents’ use of harsh punishment with 3–6 years old children.

	Never	Less than once a month	Few times in the past month	Once or twice a week in the past month	Almost everyday in the past month
Parents’ use of harsh punishment at age 3–6 years (*n* = 225)					
Swear at child	91 (40.4)	38 (16.9)	32 (14.2)	40 (17.8)	24 (10.7)
Call child names	132 (58.7)	20 (8.9)	24 (10.7)	37 (16.4)	12 (5.3)
Threaten to send child away	152 (67.6)	18 (8.0)	14 (6.2)	33 (14.7)	8 (3.6)
Slap on bottom hand arm or leg	5 (2.2)	33 (14.7)	73 (32.4)	75 (33.3)	39 (17.3)
Hit with hard object	97 (43.1)	35 (15.6)	33 (14.7)	47 (20.9)	13 (5.8)
Pinch	128 (56.9)	38 (16.9)	17 (7.6)	30 (13.3)	12 (5.3)
Hit in face, head or ears	188 (83.6)	17 (7.6)	10 (4.4)	8 (3.6)	2 (0.9)

Values are *n* (%).

### Parents’ use of harsh punishment at time 1 and child outcomes in primary school

More frequent harsh punishment at time 1 significantly predicted growth in conduct problems over time by independent observation (*p*  =  0.037), teacher (*p*  =  0.044) and parent (*p*  =  0.018) report (Table 5). More frequent use of harsh punishment by parents at time 1 also significantly predicted declining social skills from preschool to grade one of primary school by teacher (*p*  =  0.024) and parent (*p*  =  0.014) report. Frequent harsh punishment by parents during the preschool years also predicted poorer attention skills (*p*  =  0.049) but no associations were found with child impulse control (*p*  =  0.738), academic achievement (*p*  =  0.225) and oral language skills (*p*  =  0.703) (Table 5).

**Table 5.** Multilevel regression analyses showing longitudinal associations between parent's use of harsh punishment at time 1 (preschool) and child behaviour, academic achievement, oral language skills and executive function at time 2 (primary school) n  =  211 children.

	Harsh punishment			
	*B* (95% CI)	*p* value	ICC^a^	Significant covariates^b^
Observed conduct problems^1^	0.03 (0.00–0.06)^**c**^	0.037*	0.08	SES: *B* = −0.17 (95% CI −0.31 to −0.04)
Teacher-reported conduct problems^2^	1.29 (0.03–2.55)^**c**^	0.044*	0.05	None
Parent-reported conduct problems^3^	0.76 (0.13–1.39)^**c**^	0.018*	0.01	None
Teacher-reported social skills^4^	−0.70 (−1.30 to −0.09)^**d**^	0.024*	0.00	SES: *B* = 3.20 (95% CI 0.20–6.21)Child sex: *B* = 6.87 (95% CI 0.97–12.77)
Parent-reported prosocial skills^5^	−0.06 (−0.11 to −0.01)^**c**^	0.014*	0.00	Maternal involvement: *B* = 0.05 (0.01–0.1)
Oral language skills^6^	0.01 (−0.02 to 0.04))	0.703	0.00	SES: *B* = 0.16 (95% CI 0.03–0.30)
Academic achievement^7^	−0.02 (−0.04 to 0.01)	0.225	0.15	SES: *B* = 0.18 (95% CI 0.05–0.32)
Attention^8^	−0.03 (−0.05 to 0.00)	0.049*	0.04	SES: *B* = 0.22 (95% CI 0.09–0.36)
Impulse control^9^	−0.01 (−0.03 to 0.02)	0.738	0.00	None

*B* = regression coefficient.

*p < 0.05. All analyses control for child age and sex, intervention group, mothers’ education, SES, parent involvement with their child and data collector as fixed effects and school and classroom as random effects.

a Intra-cluster correlation coefficient.

b Potential covariates include child sex, SES, maternal involvement and mothers’ education.

^c^ Controlling for baseline score.

^d^ Controlling for baseline score=Preschool and Kindergarten Behaviour Scale.

^1^ Factor score of 6 child observational measures (see Table 2); ^2^Sutter–Eyberg Student Behaviour Scale; ^3^Eyberg Child Behaviour Scale; ^4^School Social Behaviour Scales 2; ^5^Strengths and Difficulties Questionnaire Prosocial Scale; ^6^sum of the standardised raw scores for story recall and following directions; ^7^factor score of letter word ID, reading comprehension, maths calculation, maths reasoning and spelling; ^8^factor score of 5 items from Preschool Self-Regulation Assessment (PSRA) rating scale: attention; ^9^factor score of 5 items from PSRA rating scale: impulse control.

### Significant covariates for child outcomes in primary school

SES, maternal education and maternal involvement with her child at time 1and child sex were controlled for in each analysis. Higher SES was associated with reduced conduct problems at school by observation (*p*  =  0.037), increased social skills by teacher report (*p*  =  0.037) and better academic achievement (*p*  =  0.007), oral language skills (*p*  =  0.017) and attention (*p*  =  0.001) in grade one of primary school (Table 5). Maternal involvement with her child was associated with increased social skills by parent report (*p*  =  0.047) and teachers reported increased social skills for girls (*p*  =  0.02).

## Discussion

This is the first longitudinal prospective study to examine the associations between harsh punishment during the preschool years and later child outcomes for children with conduct problems in LMIC. We found that higher frequency of parent's use of harsh punishment in preschool was associated with worsening child behaviour outcomes as children transition into grade one of primary school. The children were selected as being at high risk for developing conduct problems and children were scoring in the top 12.5% in their classroom for conduct problems in preschool. Higher frequency of parents’ use of harsh punishment predicted growth in child conduct problems and a reduction in child social skills at school and at home; harsh punishment also predicted poorer attention, although there were no effects on achievement, language skills and impulse control.

Parents’ use of harsh punishment with these high-risk children was pervasive: 99% of parents used physical violence and 84% used psychological aggression. This high prevalence of harsh punishment corresponds to other studies with parents of young children in Jamaica and the wider Caribbean (Lansford & Deater-Deckard, 2012; Meeks Gardner *et al*. 2008). When harsh punishment is normative within a culture, the effects on child outcomes may be less severe (Gershoff *et al*. 2010); however, in this study, we showed that even though harsh punishment is highly normative in Jamaica, frequency of harsh punishment by parents was associated with growth in conduct problems and deteriorating social skills over time for children with high levels of conduct problems at baseline. These results were robust across measurements of child behaviour using multiple informants and across home and school settings.

The children who participated in this study (1) had high levels of conduct problems and (2) were exposed to harsh punishment at baseline. Hence, we are unable to assess whether harsh punishment played a causal role in the development of children's conduct problems; we can only state that harsh punishment led to worsening behaviour over time. We also have no data to indicate whether these high-risk young children are particularly vulnerable to the negative effects of harsh punishment or whether similar associations would be evident with children without behaviour problems. This study also demonstrates the importance of operationalizing the measurement of harsh punishment in terms of frequency and/or severity, rather than using measures of prevalence or incidence, when conducting research in contexts in which harsh punishment is widely used.

Child SES in preschool was controlled for in all analyses and higher SES was associated with a fewer child conduct problems at school through observation and higher social skills by teacher report. Higher SES was also associated with children's academic achievement, language skills and attention. Poverty is a well-established risk factor for child development (Grantham-McGregor *et al*. 2007) and for mental health (Lund *et al*. 2010).

### 

#### Strengths and limitations of the study

This study has several strengths. Parents reported on their use of harsh punishment; child behaviour was measured through multiple informants including independent observations, teacher and parent report, and school achievement, oral language, child attention and child impulse control were assessed by trained research personnel. We chose well-validated and widely used teacher and parent questionnaires to measure child conduct problems and social skills. Frequency measures rather than categorical measures were used as these child behaviours fall along a continuum in the population and using frequency scores maintains the individual variability between children, increases sensitivity and reduces measurement error, especially for children with scores in a borderline range. The achievement tests were delivered in a standardised way and are likely to be more valid than using school-administered tests and more sensitive than binary and categorical measures such as grade retention, special education placement and school drop-out. All measures had good test–retest and adequate internal reliabilities, and quality control of all measures was maintained throughout the study. Attrition was low; only 6% were lost to follow-up. Several important covariates were controlled for in all analyses including child age and sex, SES, maternal education, frequency of parent involvement with child. Baseline scores for children's behaviour at school and at home were also controlled in the analyses on child behaviour.

The study also has some limitations. The small sample size limits power and there are other potential confounders that were not measured. For example, frequency of harsh punishment may covary with other risks for child behaviour problems (e.g. maternal depression, low maternal warmth and sensitivity) and children experiencing higher levels of harsh punishment may also be exposed to higher levels of other forms of violence (e.g. domestic abuse, community violence, harsh punishment at school) which are also risk factors for poor child outcomes (Baker-Henningham *et al*. 2009; Samms-Vaughan & Lambert, 2017). We had no baseline measures for child academic achievement, oral language skills and child attention and impulse control, and hence these analyses are less robust as they do not measure change in child functioning over time. Measures of child behaviour at school were conducted in the autumn term in preschool and in the summer term in grade one. It is possible that child behaviour varies across the school year and this was not controlled for in the analyses. However, all children were evaluated over the same period at both time 1 and time 2 and baseline measures were controlled in the analysis. Parents self-reported on their use of harsh punishment and they may have under- or over-reported. However, the consistency of the results suggests that the measure had reasonable validity. The measures used in this study have not been normed in the Jamaica context; however, all outcome measures have been used previously in Jamaica, have good psychometric properties and have been shown to be sensitive to change with intervention (Baker-Henningham *et al*. 2012) or sensitive to differences between children (Baker-Henningham *et al*. 2007). The sample included children with the highest level of conduct problems in community preschools situated in urban, disadvantaged areas, and hence the results are not generalisable to the general population.

#### Study implications

The high prevalence of harsh punishment by parents of young Jamaican, from this study and from previous studies (Lansford & Deater-Deckard, 2012; Samms-Vaughan & Lambert, 2017), demonstrates the urgent need for appropriate parenting interventions to train parents in alternative discipline strategies and prevent violence against children. This is important not only for moral reasons, but also because of the negative effects on child development and the associated social and economic costs (Ward *et al*. 2016). There is evidence that parenting programmes can reduce the risk of child maltreatment and improve parenting competencies (Chen & Chan, 2016); however, evidence from LMIC is limited (Knerr *et al*. 2013). Integrating such programmes into existing services would increase access and promote programme sustainability and the most common services accessed by young children are the health and education sector. In Jamaica, over 98% of young children attend preschool and hence integrating a parenting intervention to prevent harsh punishment into the preschool network is a promising approach with potential for near universal coverage. Training Jamaican preschool teachers in appropriate discipline techniques has shown benefits to teachers’ child management practices, including reductions in harsh punishment (Baker-Henningham *et al*. 2009*b*; Baker-Henningham & Walker, 2018) and a similar approach could be developed to train parents of preschool children as they enter school. Previous qualitative work with parents of preschool children in Jamaica has shown that although parents report frequent use of corporal punishment with their young child, they believe it is undesirable and ineffective (Baker-Henningham & Walker, 2009), suggesting that they would be receptive to training in behaviour management.

## Conclusion

Disadvantaged, inner city, Jamaican children identified as high risk for developing conduct problems during the preschool years were exposed to high levels of harsh punishment (defined as physical violence and psychological aggression) at home. This study presents preliminary evidence that the frequency of parents’ use of harsh punishment leads to worse behaviour trajectories over time, in terms of increased conduct problems and reduced social skills at home and at school, for these young children. The high prevalence of harsh punishment and the evidence for its negative effect on children's development demonstrate an urgent need for parenting programmes to train parents in alternative discipline strategies and to prevent violence against young children in Jamaica.

## Supplementary material

For supplementary material accompanying this paper visit http://dx.doi.org/10.1017/gmh.2018.21. click here to view supplementary material
