# Supplementary material for: Parents' use of harsh punishment and young children's behaviour and achievement: a longitudinal study of Jamaican children with conduct problems
Source: Glob Ment Health (Camb). 2018 Oct 12;5:e32. doi: 10.1017/gmh.2018.21 (PMC6236219; doi:10.1017/gmh.2018.21)
Supplement: Supplementary file 1 [file S2054425118000213sup001.docx]

**Webtable 1:** Test-Retest of Child Tests of Reading, Maths, Spelling, Language and Ratings

of Executive Function

|  | Intra-class correlation coefficient |
| --- | --- |
| Letter-word identification | 0.98 |
| Reading comprehension | 0.98 |
| Mathematical reasoning | 0.95 |
| Maths calculation | 0.99 |
| Spelling | 0.94 |
| Story recall | 0.75 |
| Following directions | 0.88 |
| Executive function | 0.85 |

Conducted with 20 children over a 2-week period

**Webtable 2:** Internal reliability and test-retest of child behaviour by teacher and parent report parents’ use of harsh punishment and involvement with child

|  | Preschool Measurements | | Primary School Measurements | |
| --- | --- | --- | --- | --- |
|  | Cronbach’s alpha | Test-retest^1^ (ICC^2^) | Cronbach’s alpha | Test-retest^3^ (ICC^2^) |
| *Teacher reported child behaviour* |  |  |  |  |
| Sutter-Eyberg Student Behaviour Inventory | 0.96 | 0.92 | 0.97 | 0.95 |
| Preschool and Kindergarten Behaviour Scales/ School Social Behaviour Scales 2 (Social Skills Scale) | 0.92 | 0.85 | 0.95 | 0.90 |
| *Parent reported child behaviour* |  |  |  |  |
| Eyberg Child Behaviour Inventory | 0.80 | 0.99 | 0.83 | 0.93 |
| Prosocial skills (Strengths and Difficulties Questionnaire) | 0.65 | 0.70 | 0.71 | 0.75 |
| *Parent reported discipline and involvement with child* |  |  |  |  |
| Parents’ use of harsh punishment | 0.64 | 0.88 | 0.69 | 0.91 |
| Parents’ involvement with child | 0.67 | 0.96 | 0.70 | 0.90 |
| *Child self-regulation skills by tester observations* |  |  |  |  |
| Child attention | - | - | 0.87 | 0.88 |
| Child impulse control | - | - | 0.85 | 0.85 |

^1^Over 2 weeks: n=20 for teacher report measures, n=18 for parent report measures

^2^ICC: intraclass correlation coefficient

^3^Over 2 weeks: n=20 for all measurements

**Webtable 3:** Factor analyses of observed child behavior in preschool and in primary school

|  | **Preschool** | **Primary School** |
| --- | --- | --- |
| **Child Observed Behaviour** | **Factor Loading** | **Factor Loading** |
| Aggression | 0.814 | 0. 800 |
| Disruptive behavior | 0.665 | 0.834 |
| Conduct problems | 0.863 | 0.824 |
| Activity | 0.783 | 0.793 |
| Follows rules | -0.909 | -0.916 |
| On-Task behaviour | -0.640 | -0.708 |
| Variance explained, % | 61.63 | 66.41 |
|  |  |  |

**Webtable 4:** Factor analysis of child academic achievement in primary school

| **Academic Achievement Tests** | **Factor Loading** |
| --- | --- |
| Letter-word ID | 0.936 |
| Reading comprehension | 0.900 |
| Spelling | 0.930 |
| Maths | 0.783 |
| Calculation | 0.794 |
| Variance explained, % | 75.92 |

**Webtable 5:** Factor analysis of Preschool Self-Regulation Assessment Rating Scale

|  | **Factor 1** | **Factor 2** |
| --- | --- | --- |
| Not distracted by sights and sounds | 0.812 |  |
| Pays attention during instructions | 0.801 |  |
| Sustains concentration | 0.797 |  |
| Doesn’t daydream | 0.786 |  |
| Careful, interested in accuracy | 0.618 | 0.415 |
| Doesn’t interrupt examiner |  | 0.800 |
| Refrains from touching test materials |  | 0.790 |
| Thinks and plans |  | 0.763 |
| No difficulty waiting |  | 0.756 |
| Remains in seat |  | 0.593 |
| Variance explained, % | 65.44 |  |
